# Of Paleo-Genes and Perch: What if an “Alien” Is Actually a Native?

**DOI:** 10.1371/journal.pone.0119071

**Published:** 2015-03-09

**Authors:** J. Curt Stager, Lee Ann Sporn, Melanie Johnson, Sean Regalado

**Affiliations:** Natural Sciences Division, Paul Smith's College, Paul Smiths, New York, United States of America; The Ohio State University, UNITED STATES

## Abstract

Documenting whether a biotic taxon is native or alien to an ecosystem has theoretical value for ecological and evolutionary studies, and has practical value because it can potentially identify a taxon as a desirable component of an ecosystem or target it for removal. In some cases, however, such background information is inadequate or unavailable. Here we use paleo-DNA to re-evaluate the historical status of yellow perch in the 6 million acre Adirondack State Park of northern New York. Yellow perch DNA in a 2200-year sediment record reveals a long-term native status for these supposedly alien fish and challenges assumptions that they necessarily exclude native trout from upland lakes. Similar approaches could be applied to other species with uncertain historical distributions and could help to identify unrecognized pockets of biodiversity.

## Introduction

Observational records are the traditional sources of information about the distributional histories of invasive species, but such records are not always available and they do not necessarily provide accurate information on past biogeography. For example, questionable status of sea lamprey (*Petromyzon marinus*) and other fishes in the northeastern United States can undermine the scientific rationales for lake and fisheries management programs [1],[2],[3]. The case of yellow perch (*Perca flavescens*) in the Adirondack uplands is noteworthy in this regard because alien status and presumed ecological incompatibility of yellow perch with brook trout (*Salvelinus fontinalis*) populations are used to justify controversial piscicidal treatments of Adirondack lakes [4],[5], largely on the basis of surveys that failed to detect perch during the late 19th century [6].

**Fig 1:**
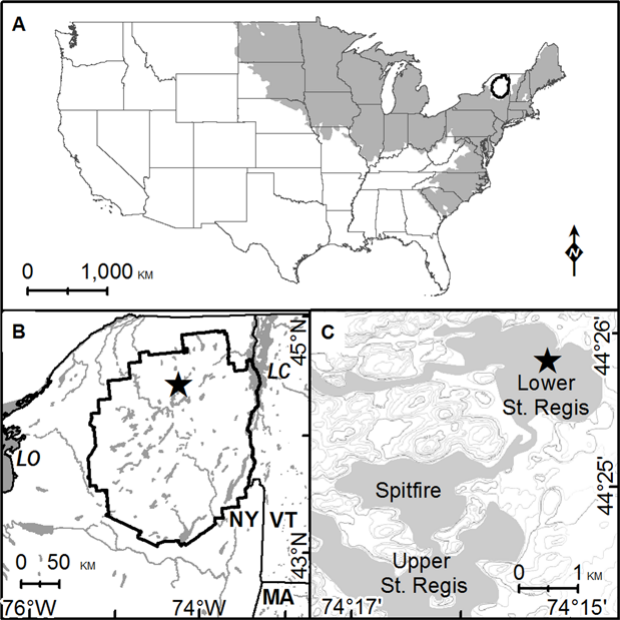
Site maps. A. Approximate range of yellow perch in the United States shown in gray (after reference 8). Note the prominent gap in northern New York, with the Adirondack Park boundary superimposed in bold. Scattered relict populations along the Florida coast (reference 33) not shown. B. The Adirondack State Park, NY. Lower Saint Regis Lake indicated by star. LC = Lake Champlain. LO = Lake Ontario. C. Saint Regis chain of lakes in Paul Smiths, NY. Coring site indicated by star.

Yellow perch are widely distributed in the northeastern United States and eastern Canada over a broad range of elevations and diverse lake and stream habitats (Fig. 1A) [3],[7],[8]. However, the position of state and local fisheries scientists, management organizations, and environmental groups has been that perch were introduced to the Adirondack uplands during the last century or so [6],[9],[10],[11]. A hitherto overlooked report of yellow perch in Lake Sanford in the central Adirondacks in 1863 [12] does not by itself necessarily establish native status because sporadic stocking of warm-water fishes occurred in the region during the early 19^th^ century [6],[10]. However, the excellent dispersal capability and wide distribution of yellow perch in eastern North American lakes and streams also makes their supposed former absence from the Adirondack uplands suspect and worthy of re-examination [13]. In this context, the biogeographic history of yellow perch is of more than academic interest because the presumption of alien status influences fisheries management policy in the roughly 3000 lakes and ponds of the Adirondack State Park (Fig. 1B).

Eradication of invasive fish by treatment with rotenone or toxaphene (reclamation) has been conducted in more than 100 Adirondack lakes since the 1950s by the New York State Department of Environmental Conservation (NYSDEC) despite opposition from various state and local organizations [4],[5],[14]. Although different stakeholders disagree over the use of reclamation in the region, all parties have assumed that yellow perch are not native to the uplands. We address that assumption here by examining environmental DNA (eDNA) in sediment cores from a lake in the north central Adirondacks. Our findings challenge two common assumptions about yellow perch in this region: that they are non-native to the uplands and that they do not coexist with native brook trout. We also suggest that eDNA can be used in similar fashion to inform conservation and management programs elsewhere.

The focus of this study was Lower Saint Regis Lake in Franklin County, NY (Fig. 1B,C; 44°25’54” N, 74°16’15” W; 494 m amsl; max depth 11.6 m) the lowest in a chain of mesotrophic, circumneutral lakes in the St. Lawrence River watershed within the northern Adirondack uplands. A biological survey in 1930 reported no yellow perch but abundant brook trout in the St. Regis lakes [5]. Today, the fish community is instead dominated by yellow perch, northern pike (*Esox lucius*), largemouth bass (*Micropterus salmoides*), and golden shiner (*Notemigonus crysoleucas*). Local residents report that brook trout were common in Lower St. Regis Lake during the 1960s and early 1970s, but absent since the late 1970s. Yellow perch were caught in the lake during the 1970s, as well, but their numbers increased notably during a period of cultural eutrophication in the 1960s and 1970s that also saw the establishment of largemouth bass and northern pike in the lake along with the disappearance of brook trout.

We examined the history of yellow perch in Lower Saint Regis Lake by analyzing eDNA specific for yellow perch in sediment core samples. Such eDNA can remain suspended in lakes long after being shed or following decomposition [15], and has been shown to persist as "paleo-DNA" for centuries to millennia in aquatic sediments [16],[17],[18],[19],[20],[21].

Recently released eDNA in lake and river water has been used to document the presence of invasive Asian carp (e.g. *Hypophthalmichthys molitrix*) in the Great Lakes region [15], and similar methods using paleo-DNA have been used to reconstruct long-term histories of copepods [17], penguins [18], and various other taxa [16],[19],[20],[21]. Here we use paleo-DNA to document the long-term history of a North American aquatic vertebrate and challenge its "invasive" classification.

## Materials and Methods

### Ethics statement

No specific permissions were required for coring on the Adirondack lakes (this is a state park but doesn't require research permits for these lakes); appropriate research permit was obtained from the Tanzanian government for coring on Lake Tanganyika was obtained before the core was collected for a previous study, described in citation [27]. The perch was not sacrificed for the purpose of this study but was taken legally as a game fish by an angler for personal use as food. We collected a piece of fin tissue after the fish was already deceased. The fish was dispatched with a sharp blow to the head with a hard object, as is standard procedure among local anglers.

### Sediment and microfossil analyses

A 35 cm gravity core was obtained from the center of Lower St. Regis Lake and extruded vertically, and sediment subsamples were taken at 5 cm intervals from the interior of the core for initial screening for presence of yellow perch eDNA. A 135 cm piston core was then taken from a nearby site within the same lake (Fig. 1C). An accelerator mass spectrometry (AMS) date from bulk organic sediments at 125 cm depth in the piston core yielded an age of 2195 ± 29 radiocarbon years before present (DAMS-002216), with a calibrated 2-sigma age range of 2131–2315 calendar years before present (CALIB 6.0) [22] that is consistent with age-depth profiles from other lakes in the region [14],[23],[24]. The granitic bedrock and soils in the watershed do not cause hard-water effects that might produce spuriously old radiocarbon ages, and the inferred timing of changes in the microfossil record further supports a millennial-scale basal age (see below).

**Fig 2:**
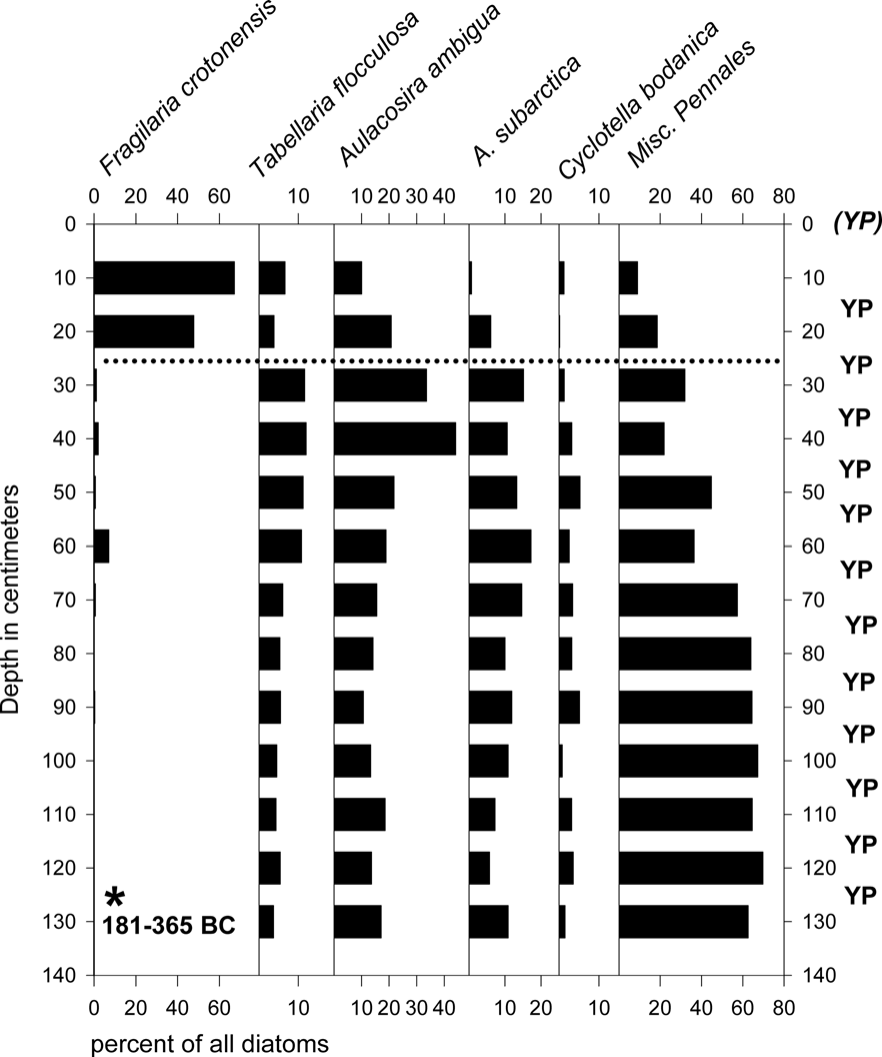
Diatom stratigraphy of sediment core from Lower Saint Regis Lake. Increased *Fragilaria crotonensis* percentages above the dotted line represents cultural eutrophication in the mid-20^th^ century and indicates reasonable stratigraphic integrity down-core. Asterisk: calibrated AMS date. YP = sample tested for yellow perch eDNA; all were positive. (YP) = positive yellow perch eDNA sample from modern mud-water interface in an adjacent gravity core.

Fossil diatom assemblages were used to document the intact nature of the stratigraphy. Increased relative abundances of *Fragilaria crotonensis* in the upper 20 cm of the piston core (Fig. 2) represent the recent eutrophication of the lake. This taxon is commonly associated with eutrophication in the Adirondacks [14], and it was the most abundant diatom in the plankton during the 1970s when biogenic clouding of the water column produced mean Secchi depths as low as 1.5 m [25]. Although a precise onset date for this change in the diatom assemblages was not determined from the core, its clear presence near the top of the sequence (as well as the top of the gravity core that captured the mud-water interface) shows that the sediments in this core were not heavily disturbed in a way that would have introduced significant recent material into the deepest portions of the core.

Care was taken to avoid contamination of the sediments with modern yellow perch DNA in the field and laboratory [26]. The pristine barrel of the piston core was sawed open longitudinally and sampled at 10 cm increments in a dedicated Class II biosafety cabinet under negative air pressure. Sterile spatulae were used to extract sediment samples from the center of the column in order to avoid peripheral sediments that might have undergone frictional mixing during coring. In addition, multiple controls were used to ensure that results did not result from contamination both in the field and in the laboratory [26]. For control amplifications, sediments were sampled at the same time from the tops of cores from a local ephemeral vernal pool and two lakes from which yellow perch are known to be absent: Wolf Lake, NY [23], and Lake Tanganyika, East Africa [27]. Multiple “no template” controls were included in all PCR amplifications.

### Paleo-DNA analyses

A 124 base pair portion of the mitochondrial (my) DNA cytochrome oxidase subunit 1 (CO1) gene was chosen as a species-specific barcode marker for yellow perch [28]. This traditional DNA barcode region is approximately 650 base pairs, but a smaller amplicon size was selected because DNA degradation may have interfered with detection of the full-length barcode region and because small amplicons within the CO1 gene have been shown to be effective in species identification of fishes [29].

*Perca flavescens*-specific primers were developed using a published sequence of yellow perch CO1 gene from a voucher specimen [30] and software (Primer Blast function) available on the National Center for Biotechnology Information (NCBI) website (www.ncbi.nlm.nih.gov). Species specificity was verified (screening for unintended targets) using the Primer Blast function. The DNA barcode region [28] of the CO1 gene from approximately 30,000 fish species is contained within the NCBI database [31]. While no unintended targets of concern were found using *in silico* testing of the primer pair chosen, DNA sequencing of amplicons produced from environmental samples was conducted to verify authenticity, as described below.

### DNA purification and amplification

Samples of packed sediment, collected at 10 cm increments throughout the longer core and from the top and bottom of the gravity core, were prepared by centrifuging at 5000 x g in a microfuge. Packed sediments (250 mg) were subjected to DNA purification using the PowerSoil DNA purification kit (MoBio Laboratories) following manufacturer’s instructions. Total DNA recovered from samples was quantitated using a NanoDrop spectrophotometer. Surface sediments yielded 23 ng/μl, and deeper sediments yielded between 1 and 7 ng/μl total DNA. Total genomic DNA from a locally-collected yellow perch was also prepared using the DNeasy Blood and Tissue Kit, Qiagen (Netherlands).

**Fig 3:**
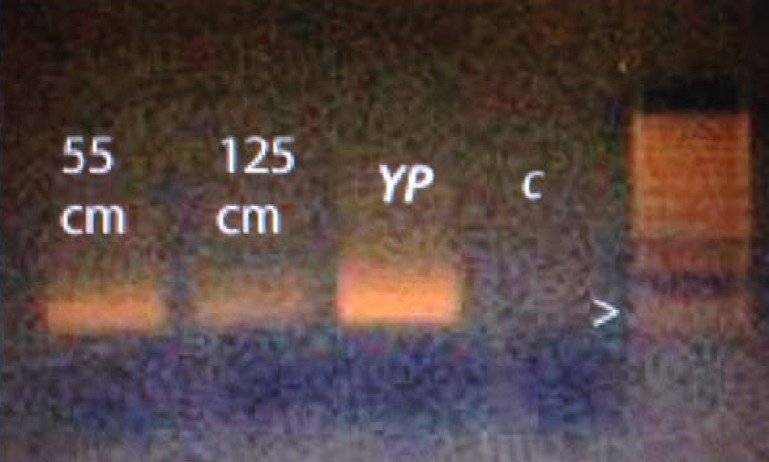
Representative results of gel electrophoresis of amplified DNA. Shows the presence of the yellow perch barcode sequence (CO1) in sediment samples from 55 cm and 125 cm depths in the Lower Saint Regis core. YP = Yellow perch barcode. C = No-template control lane. Arrow indicates 100 base pair position on molecular ruler lane.

Primer sequences used for species-specific amplification of yellow perch eDNA were as follows: yellow perch (124 base pair amplicon) forward primer: CCT TGT TCG TAT GGG CTG TA, reverse primer: GCA GGA TCG AAG AAA GTG GT. Polymerase Chain Reaction (PCR) was carried out using standard PCR mastermix from 5 PRIME (Gaithersburg, MD), containing 5 pmoles of each primer per 10 μl reaction volume. For deeper sediments, one microliter of purified DNA was used per 10 μl reaction, except surface sediments which were diluted to contain 10 ng of DNA per 10 μl reaction. Cycling conditions were optimized using total genomic DNA from yellow perch. Cycling protocol was as follows: 94°C for 1 min, followed by 5 cycles of 94°C for 30 sec, 48°C for 1.5 min, and 72°C for l min, then 35 cycles of 94°C for 30 sec, 50°C for 1.5 min, and 72°C for 1 min, then a final extension of 72°C for 4 min. Products were analyzed on 2% agarose gels stained with ethidium bromide and photographed under ultraviolet illumination (Fig. 3).

### Cloning and sequencing

To verify the authenticity of yellow perch DNA, amplicons were cloned using traditional TA cloning methodology prior to sequencing. Amplicons prepared from the piston core samples by PCR (without further purification) were ligated into pCR8/GW/TOPO vectors using the pCR8/GW/TOPO Cloning Kit (Invitrogen, Carlsbad, CA) and used to transform One Shot Chemically Competent *E*. *coli* (Invitrogen, Carlsbad, CA). Colonies obtained on Luria-Bertani medium under spectinomycin selection (100 micrograms/ml) were screened for presence of inserts by PCR using a direct colony screening method. PCR reactions were assembled and cycled as described above, but small amounts of bacterial templates from each colony were directly added using a sterile pipette tip. Thirty bacterial transformant colonies were screened, of which 29 were found to contain an insert of the expected size (124 bp) as determined by gel electrophoresis. The remaining single colony was found to contain only vector sequence and likely arose by vector self-ligation. Plasmids were purified from overnight cultures of three colonies under spectinomycin selection and were submitted to the Cornell University Institute of Biotechnology for sequencing using an Applied Biosystems Automated 3730xl DNA Analyzer, Big Dye Terminator chemistry, and AmpliTaq-FS DNA Polymerase using M13 forward sequencing primer.

**Fig 4:**
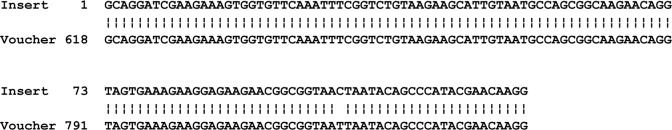
Sequence alignment. Cloned insert with yellow perch mitochondrial cytochrome oxidase subunit 1 (GenBank voucher PEFL-ON-BLAC-4–3).

Sequencing of the cloned inserts revealed 99% identity with that of the voucher specimen (Fig. 4). A single substitution (T to C) was seen in two of the three inserts sequenced at position 101 of the amplicon, indicating either the presence of a polymorphism at this site or a chemical change in the pyrimidine base as has been reported in other ancient DNA samples [16]. All data used in this paper are available upon request (contact first author, JCS).

## Results

All samples in the two cores from Lower Saint Regis Lake tested positive for yellow perch paleo-DNA (Figs. 2, 3, and 4). None of the samples from the yellow perch-free vernal pool or lakes tested positive. In combination with the AMS and diatom evidence for the intact nature of the Lower Saint Regis sediment sequence, we conclude that these results accurately document the presence of yellow perch paleo-DNA during the time frame represented by the core. Together, these results show that yellow perch were present in the Saint Regis watershed for at least two millennia, more than enough time to be considered native to the Adirondack uplands. Furthermore, the presence of perch paleo-DNA at multiple depths in the core also suggests that this species is likely to have coexisted with brook trout long before the recent onset of eutrophication.

Further studies of additional lakes will be required to determine the full historical distribution of yellow perch in the Adirondacks. The lack of perch paleo-DNA in the Wolf Lake core is consistent with observational records from that site [23], and it supports earlier observations that yellow perch were not universally distributed among Adirondack lakes [6],[10]. Nonetheless, our findings raise important questions regarding the ecology and management of yellow perch in the Adirondack Park.

## Discussion

It is commonly stated in fisheries literature for the Adirondack region that yellow perch are nuisance fish that exclude brook trout when they colonize a lake by competing for food and spawning sites, consuming the eggs of other fishes, and other processes [5],[10]. According to one biological survey, “…the one outstanding reason why so many … Adirondack areas are now unfit for the native species is that… yellow perch… and other species of non-native warm water fishes have been introduced" [9]. Although colonization of brook trout habitat by yellow perch may indeed result in competitive exclusion under certain conditions, our results from Lower Saint Regis Lake show that yellow perch coexisted with brook trout in that lake during the early 20^th^ century and probably for many centuries before that, as well. This suggests that the causes of declines in local brook trout populations may need to be re-evaluated. Recent introduction of northern pike and largemouth bass, overfishing, stocking with hatchery-raised fish, climate change, and water quality declines may represent more direct challenges to brook trout in some cases, and they could also offer a competitive edge to yellow perch which are more likely to thrive in warmer, more productive waters than brook trout [14],[32].

Changing the status of species such as yellow perch from alien to native can encourage new approaches to the conservation of biodiversity. A major objective of fisheries management by the NYSDEC is to preserve endemic Adirondack strains of brook trout, which is a primary justification for eradicating perch from potential spawning lakes [4],[5],[10]. However, because yellow perch have inhabited the uplands for thousands of years they, too, might have diverged into endemic sub-populations there as has been documented elsewhere [30],[33]. Future investigations into the genetics of yellow perch in the Adirondacks might therefore identify a hitherto-unrecognized pool of genetic diversity with aesthetic, scientific, and/or recreational value that may also face pressing environmental threats. Recent declines of yellow perch populations are causing concern in the Great Lakes, for example, and this species can be surprisingly sensitive to competition or predation, water quality degradation, and climate change [34],[35].

In light of the historical and paleo-DNA evidence which document the native status of yellow perch in the Adirondack uplands as well as the unique genetic composition of subpopulations of this species in the adjacent Champlain basin (Fig. 1B) [30],[33], we suggest that a more nuanced approach to the management and conservation of this species may be advisable. Similar use of paleo-DNA holds great potential to address other unresolved questions in aquatic ecology as well, including the evolution and dispersal of taxa, the relative effects of "top-down" and "bottom-up" trophic cascades on lake productivity, and the contribution of acid deposition to the distribution of fishless lakes in acid-sensitive regions.
